# Advances in artificial intelligence and thermal analysis for brain tumor detection: a review of models, methods, and modalities

**DOI:** 10.3389/frai.2026.1744410

**Published:** 2026-03-30

**Authors:** Abedalmuhdi Almomany, Uzair Soomro, Anwar Al Assaf, Alaa Abd-Alrazaq, Rafat Damseh, Muhammed Sutcu, B. S. Ksm Kader Ibrahim, Barış Yıldız

**Affiliations:** 1 Department of Electrical and Computer Engineering & Applied Innovation Research Centre (GEAR), Gulf University for Science & Technology, Hawally, Kuwait; 2 Department of Computer Engineering, Hijjawi Faculty for Engineering Technology, Yarmouk University, Irbid, Jordan; 3 Aircraft Maintenance Department, Faculty of Aviation Sciences, Amman Arab University, Amman, Jordan; 4 AI Center for Precision Health, Weill Cornell Medicine-Qatar, Doha, Qatar; 5 College of Information Technology, Al Ain, United Arab Emirates; 6 Industrial Engineering Department, Atilim University, Ankara, Türkiye

**Keywords:** artificial intelligence, bioheat transfer models, CNNs, Computed Tomography, convolutional neural networks, CT, deep learning, hybrid architectures

## Abstract

Brain tumors pose a major challenge in neuro-oncology due to their high mortality rates and complex diagnosis. This review summarizes recent advances in using artificial intelligence (AI), particularly deep learning, in conjunction with thermal imaging and simulated thermal mapping for brain tumor detection. AI methods such as convolutional neural networks (CNNs), hybrid architectures, and bioheat transfer models, including the Pennes equation, are evaluated to determine how temperature variations, tumor biology, and image preprocessing influence malignancy classification. Traditional imaging techniques, such as Magnetic Resonance Imaging (MRI) and Computed Tomography (CT), provide detailed structural information but are often costly, invasive, and limited in their ability to capture physiological data. Recent studies indicate that integrating AI with thermal imaging, either through direct infrared thermography or simulated thermal maps derived from MRI, enables non-invasive, physiology-aware diagnosis. The review examines current approaches to thermal data preprocessing, simulation, deep learning-based tumor segmentation, and malignancy prediction, as well as key evaluation metrics, model interpretability tools, and recent performance outcomes. Despite ongoing progress, challenges remain, including limited availability of multimodal datasets, variability in thermal signatures, and the need for clinical validation. Future research directions include large-scale data collection, advanced thermal modeling, multimodal fusion frameworks, and the development of explainable AI tools that meet clinical standards. In resource-limited settings, AI-powered thermal imaging may serve as a valuable supplement to traditional diagnostics, offering safer, more precise, and more accessible brain tumor detection. This technology has the potential to improve patient outcomes and transform neuro-oncology practices by integrating anatomical and functional insights. This review critically evaluates current evidence and identifies the challenges that must be addressed to facilitate the translation of promising research into clinical practice.

## 1 Introduction

**Figure 1:**
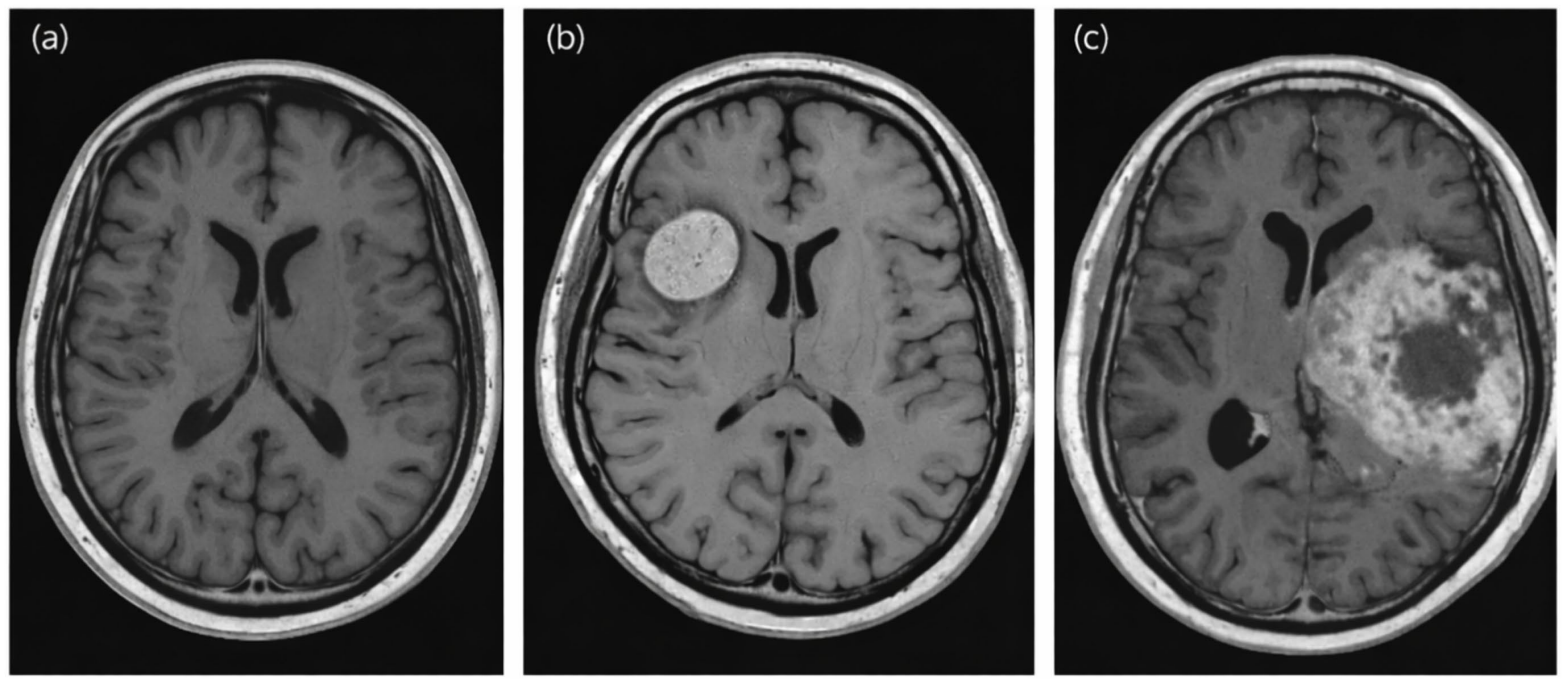
Representative MRI images of normal brain tissue **(a)**, benign tumors **(b)**, and malignant tumors **(c)**. Normal anatomy is symmetric without focal lesions. Benign tumors appear well circumscribed with minimal edema. Malignant tumors typically exhibit irregular, infiltrative margins, heterogeneous signal intensity, and mass effect (Karagoz et al., 2024). Licensed under the terms and conditions of the Creative Commons Attribution (CC BY) license (https://creativecommons.org/licenses/by/4.0/).

Brain tumors, whether benign or malignant, are abnormal cell growths in the brain (Figure 1) that can severely disrupt neurological function because the brain is a confined organ (Aleid et al., 2023). There are over 150 different types of these tumors, and they cause many neurological problems and deaths worldwide (Aleid et al., 2023; Kaifi, 2023; Berghout, 2025). It is crucial to identify the issue as early as possible, especially with malignant lesions like glioblastomas, which grow quickly and spread to nearby brain tissue (Li, 2025). Population-based data from the Central Brain Tumor Registry of the United States (CBTRUS) indicate that glioblastoma, the most common malignant primary brain tumor, has a median survival of approximately 8 months and a five-year relative survival rate of 6.9%. These statistics emphasize the aggressive nature of glioblastoma and demonstrate the urgent need for enhanced diagnostic strategies and prompt clinical intervention (Ostrom et al., 2022).

MRI and CT are the two primary imaging modalities used to detect brain tumors. MRI provides excellent soft-tissue contrast and can capture images of multiple parameters simultaneously, enabling detailed visualization of tumor shape, swelling, and necrotic cores (Krishnan et al., 2024; Albalawi et al., 2024). CT scans are typically used in emergency cases because they are quick and easy to get. Still, they do not provide sufficient detail in soft tissues and expose patients to ionizing radiation (Ahmed et al., 2024). MRI and CT scans are essential in medicine, primarily used to reveal the structure of organs and tissues. To confirm the results, histopathological biopsies are often needed. Additionally, their high cost and infrastructure requirements make them inaccessible, particularly in low-income areas (Yurtsever et al., 2024; Ullah and Kim, 2025).

At the same time, significant advances have been made in the use of artificial intelligence (AI) and deep learning (DL) for medical image analysis. CNNs and transformer-based models are the most effective at identifying and classifying brain cancers from MRI data (Karagoz et al., 2024; Zeineldin and Mathis-Ullrich, 2023; Wang et al., 2021). Alongside these improvements, thermal imaging, particularly infrared thermography (IRT), has gained popularity as a noninvasive, complementary method. IRT records temperature changes on or near the brain surface, which are indicative of physiological changes, such as aberrant blood flow and increased metabolic activity (Rezaei, 2025; Chaichana and Quiñones-Hinojosa, 2019; Raghavapudi et al., 2021). These thermal patterns are useful because cancerous tumors typically exhibit distinct patterns, such as hot spots associated with angiogenesis and cold spots associated with necrosis (Cleveland Clinic, 2023; Bousselham et al., 2018). Furthermore, recent studies have investigated simulated thermal mapping, in which bioheat transfer models, such as Pennes’ equation, are applied to grayscale MRI data to generate pseudo-thermal images (Knapp et al., 2022; Ring and Ammer, 2012). These simulated maps integrate physiological data with structural imaging, allowing AI models to analyze multimodal inputs and enhance diagnostic accuracy (Civilibal et al., 2023; Wahab et al., 2015).

This review aims to give a thorough overview of the emerging field of AI-driven thermal imaging for brain tumor detection. It discusses recent advances in preprocessing, simulation techniques, deep learning architectures, and malignancy prediction based on thermal patterns. The review also considers performance evaluation metrics and identifies key future research directions. By combining anatomical and physiological data, AI-enhanced thermal imaging has the potential to provide more accurate, non-invasive, and cost-effective brain tumor diagnostics. This review specifically addresses: The role of AI in analyzing thermal and pseudo-thermal images for tumor detection.

- Techniques for converting grayscale MRI into thermal or RGB-enhanced formats.
- The use of bioheat models (e.g., Pennes’ equation) for simulating intracranial temperature distributions.
- Performance evaluation of AI models for tumor segmentation and malignancy prediction.
- Existing challenges and future directions for clinical adoption and translational research.

Unlike previous reviews that have primarily examined either AI-based MRI analysis or thermography as standalone modalities, this review addresses the limited exploration of integrating artificial intelligence with both real infrared thermography and MRI-derived thermal simulation for brain tumor diagnostics. Rather than focusing solely on algorithmic performance, it establishes a unified framework that links AI outcomes to tumor-related physiological alterations and critically evaluates validation gaps, dataset limitations, and practical challenges that affect clinical translation and implementation.

This study uses a narrative review framework to synthesize and contextualize existing research on thermal imaging and computational modeling approaches relevant to brain tumor evaluation. The literature review prioritized peer-reviewed journal publications that offer experimental, clinical, or theoretical insights, while selectively including conference proceedings that introduced novel methodological perspectives or early proof-of-concept results not yet represented in archival journals. The review encompasses recent studies, thereby reflecting the field’s state at the time of manuscript preparation. The selected works were critically examined and integrated to distinguish between validated evidence and exploratory research and to identify prevailing challenges and open research directions.

## 2 Imaging modalities and tumor physiology

**Figure 2:**
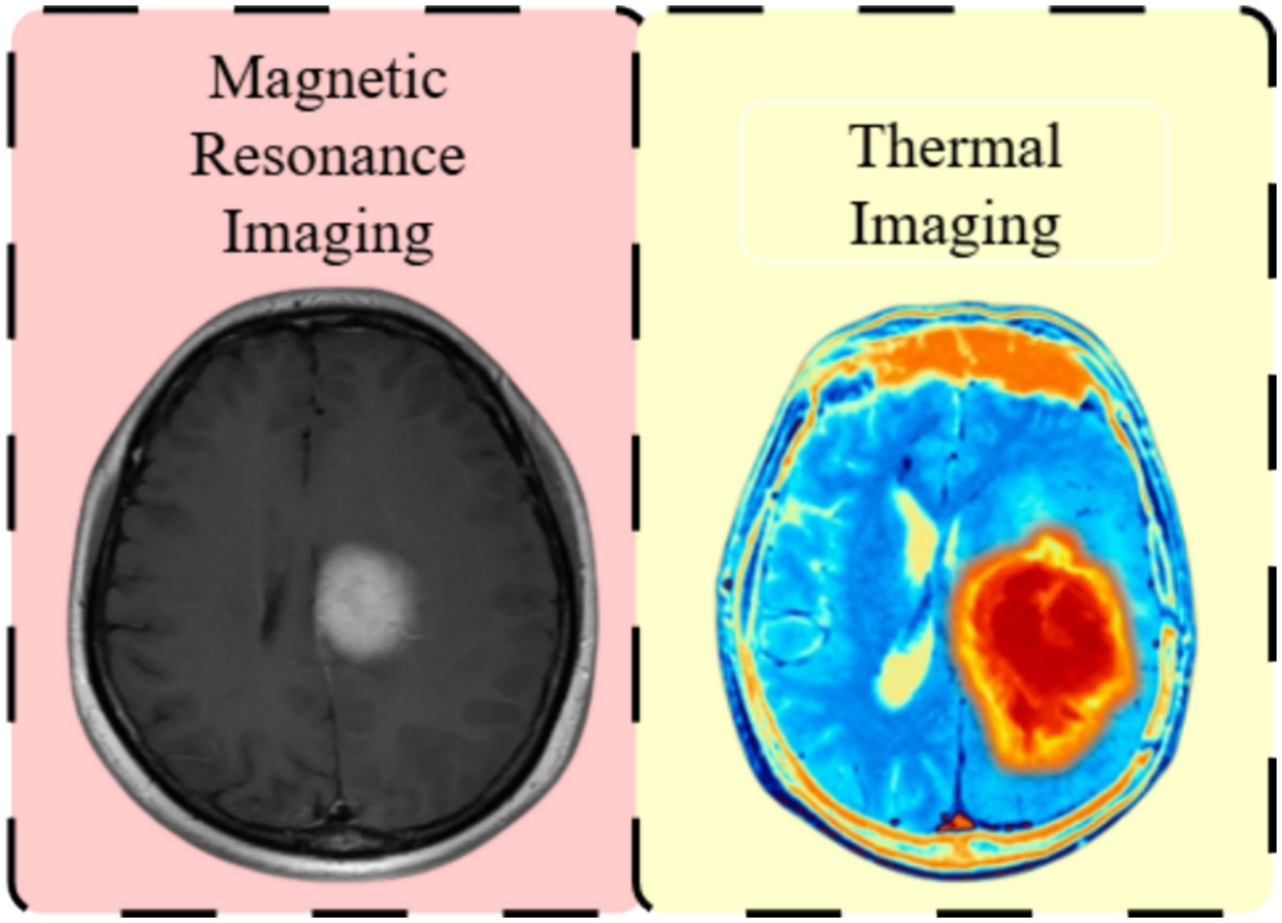
Structural MRI versus thermal imaging for brain tumor analysis. MRI depicts anatomical features, whereas thermal imaging reveals physiological abnormalities associated with tumor activity (Appiah et al., 2024).

MRI remains the gold standard for brain imaging because it provides superior anatomical detail. However, it does not capture thermal or metabolic data, which are crucial for understanding tumor behavior, as shown in Figure 2. This figure offers a conceptual comparison between conventional structural MRI and thermal imaging in brain tumor evaluation. It illustrates a malignant brain tumor at a typical clinical stage, chosen to highlight key anatomical and physiological differences rather than to specify a tumor grade. MRI mainly highlights structural features like tumor boundaries, edema, and mass effect, while thermal imaging detects physiological changes such as abnormal metabolism and blood perfusion (Appiah et al., 2024). This distinction emphasizes how thermal imaging complements anatomical imaging by enhancing tumor characterization. Specifically, infrared thermography (IRT) provides physiological insights by measuring surface or tissue temperature, as tumors often display thermal anomalies due to increased metabolic activity or altered blood flow, making thermal analysis a valuable addition to traditional diagnostics.

### 2.1 Conventional imaging for brain tumor diagnosis

MRI is considered the most effective method for evaluating brain tumors because of its excellent soft-tissue contrast and ability to provide detailed anatomical and functional information through various sequences, including T1-, T2-, and FLAIR-weighted, diffusion-weighted, and contrast-enhanced sequences (Krishnan et al., 2024; Albalawi et al., 2024). MRI effectively delineates tumor margins, detects peritumoral edema, and assesses treatment response (Albalawi et al., 2024). Computed Tomography (CT) is often used in emergency situations because of its speed and widespread availability. However, it is limited by reduced soft-tissue contrast and exposure to ionizing radiation, making it less suitable for the comprehensive characterization of brain tumors (Ahmed et al., 2024; Yurtsever et al., 2024). Both MRI and CT require expert interpretation, and diagnostic accuracy may be affected by radiologists’ experience and workload (Ullah and Kim, 2025).

In CT imaging, tissue characterization depends on X-ray attenuation values measured in Hounsfield Units (HU), which help distinguish normal brain tissue, edema, hemorrhage, and calcifications. Malignant tumors often appear hypodense or show mixed density, while acute hemorrhages typically look hyperdense. Intravenous iodinated contrast agents improve detection by highlighting blood–brain barrier disruption and tumor blood supply. Similarly, MRI employs gadolinium-based contrast agents, especially in T1-weighted sequences, to better define tumor margins and reveal malignancy-related enhancement patterns. Although these contrast agents increase sensitivity, accurate diagnosis still heavily relies on radiological expertise and clinical information.

### 2.2 Surface infrared thermography versus intraoperative thermography

**Figure 3:**
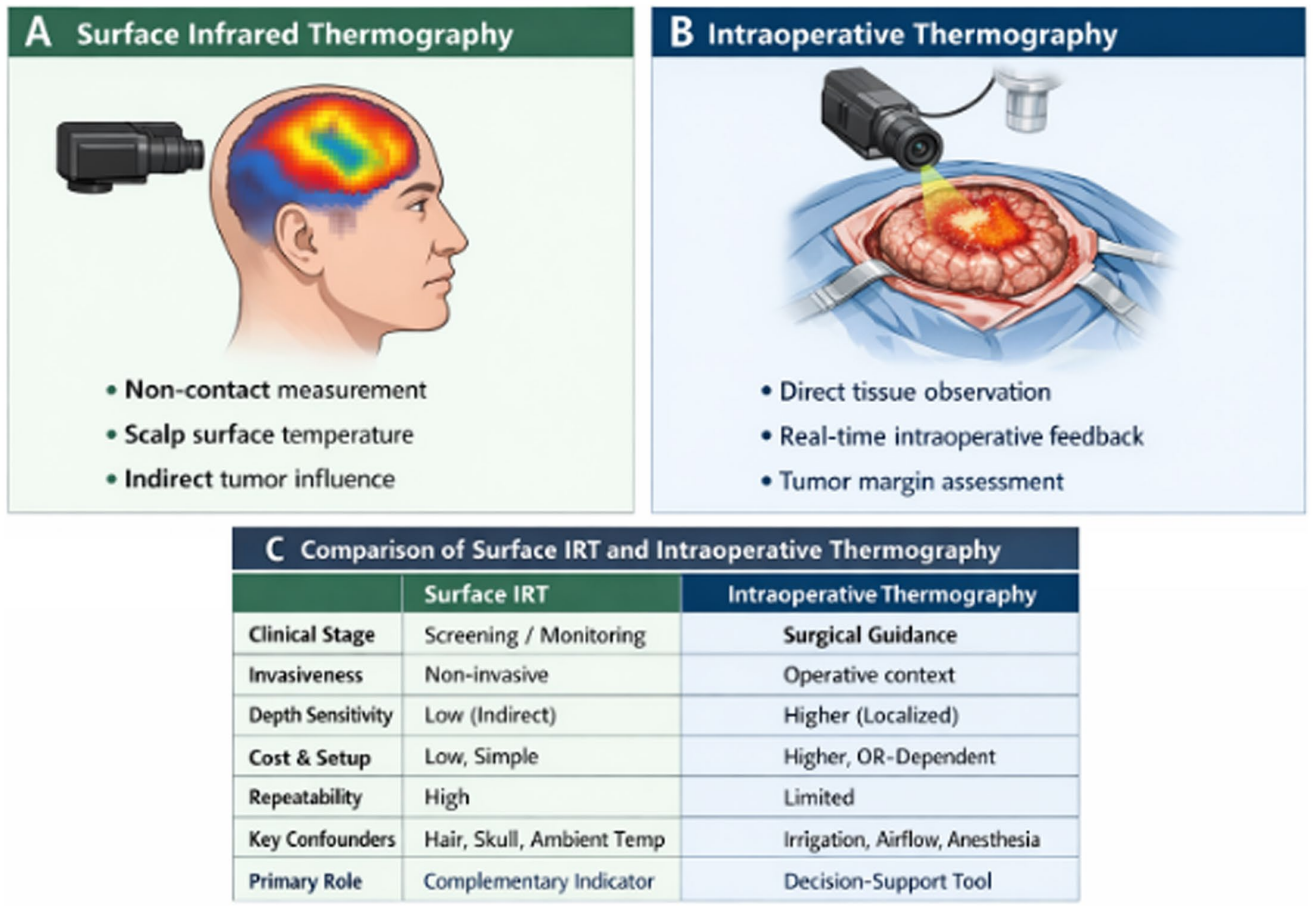
Comparison of surface infrared thermography and intraoperative thermography in brain tumor assessment, highlighting their acquisition setups, clinical roles, and key advantages and limitations.

Tumor-induced hypermetabolism and angiogenesis produce localized thermal anomalies, providing the physiological basis for the use of infrared thermography in brain tumor assessment (Rezaei, 2025; Chaichana and Quiñones-Hinojosa, 2019). Thermographic applications are generally categorized as surface infrared thermography (surface IRT) and intraoperative thermography, distinguished primarily by acquisition conditions and clinical context. Surface IRT is a non-contact method that measures scalp temperature distributions and can reveal thermal asymmetries linked to altered intracranial metabolism and blood flow. However, these measurements are indirect indicators of underlying pathology because of signal attenuation through the scalp and skull. Intraoperative thermography, by contrast, is performed during neurosurgical procedures after the brain or tumor region has been exposed. This approach enables localized, real-time observation of temperature variations within the surgical field, potentially providing additional information about tissue perfusion and tumor boundaries. Figure 3 illustrates the conceptual distinction between surface IRT and intraoperative thermography, highlighting their distinct clinical applications and complementary roles in brain tumor evaluation (Raghavapudi et al., 2021).

### 2.3 Thermal imaging and tumor physiology

Conventional neuroimaging modalities, such as Computed Tomography (CT) and magnetic resonance imaging, are essential for detecting, localizing, and characterizing brain tumors (Bruzzone et al., 2012). Although these techniques provide detailed anatomical and, in some cases, functional information, they primarily reveal morphological changes rather than the physiological and metabolic processes underlying tumor progression (Amyot et al., 2015). Brain tumors often exhibit altered cellular metabolism and abnormal angiogenesis, resulting in changes in blood flow, heat generation, and thermal dissipation within affected tissues. Recognizing these physiological alterations underscores the need for complementary imaging approaches, such as infrared thermography, which aim to capture functional signatures of tumor activity.

Table 1 presents the levels of evidence supporting the use of surface infrared thermography, intraoperative thermography, and simulated thermal imaging for brain tumor evaluation. Although experimental thermographic approaches are supported by a limited number of observational studies, simulation-based methods are primarily theoretical and lack direct clinical validation. This comparison delineates the current maturity of evidence among thermographic modalities and underscores the need for further validation.

**Table 1:** A comparative analysis of evidence maturity and validation levels for surface infrared thermography, intraoperative thermography, and simulated thermal imaging in brain tumor applications.

Approach	Primary evidence type	Level of evidence maturity	Typical study scale	Clinical validation status	Key notes / limitations	Ref
Surface infrared thermography (IRT)	Experimental and observational clinical studies	Moderate (well-established physiological basis; limited diagnostic specificity)	Small to medium patient cohorts	Investigational / complementary to conventional imaging	Indirect measurements; thermal signals attenuated by scalp and skull; sensitive to environmental and physiological factors	((Rezaei, 2025); (Chaichana and Quiñones-Hinojosa, 2019); (Raghavapudi et al., 2021)),
Intraoperative thermography	Intraoperative observational studies and case series	Low–Moderate (procedure-specific and context-dependent evidence)	Small cohorts and case reports	Exploratory clinical use during surgery	Limited to operative settings; influenced by irrigation fluids, airflow, anesthesia, and timing of acquisition	((Chaichana and Quiñones-Hinojosa, 2019); (Raghavapudi et al., 2021))
Simulated/computational thermal imaging	Numerical modeling and bioheat transfer simulations	Low (proof-of-concept and theoretical evidence)	Simulation-based studies	No direct clinical validation	Highly dependent on modeling assumptions, tissue parameters, and boundary conditions	(Das et al., 2013)

### 2.4 Simulated thermal maps derived from MRI

Direct intracranial thermal measurement is difficult; therefore, simulated thermal maps are used to estimate temperature distributions within brain tissue. By applying bioheat transfer models, such as Pennes’ equation, to MRI intensity values, researchers predict spatial temperature variations that reflect tissue metabolism and perfusion (Knapp et al., 2022; Ring and Ammer, 2012). Based on our review, while pseudo-thermal maps allow physiological modeling without specialized equipment, they may not fully capture the complex thermal dynamics of live tissue. This limitation is especially evident in heterogeneous tumor environments, where simulated values might underestimate perfusion-related cooling effects. When validated against intraoperative thermography or thermometry, simulated maps have shown a strong correlation with measured temperature patterns (Bousselham et al., 2018; Civilibal et al., 2023).

### 2.5 Multimodal integration

Integrating structural and functional imaging data has become an increasingly active area of research. Combining MRI with real or simulated thermal data enables AI models to learn richer feature representations, thereby improving sensitivity and specificity in tumor detection and classification (Karagoz et al., 2024; Wahab et al., 2015; Vardasca et al., 2019). Research shows that using multiple medical imaging modalities, such as MRI, PET, spectroscopy, and thermal imaging, can improve diagnostic accuracy. For example, the BRATS challenge has shown that combining MRI scans improves tumor edge delineation. However, few studies have examined the use of thermal imaging alongside these methods, and we still need more evidence to link this combination to clinical outcomes. Studies have shown that multimodal CNNs trained on MRI and thermal inputs outperform single-modality models, underscoring the potential of thermal imaging to enhance conventional diagnostics (Das et al., 2013; Fernández-Ovies et al., 2019; Isensee et al., 2021).

From our perspective, integrating structural MRI with physiological thermal data represents a meaningful step toward functionally grounded neurodiagnostics, moving beyond anatomy-centered evaluation. Despite the currently limited scale of thermographic evidence, its biological foundation and complementary potential support the need for systematic validation. We therefore encourage collaborative efforts to design and conduct studies that harmonize physiological modeling frameworks with rigorously acquired clinical datasets.

## 3 Thermal image processing and simulation

Image preprocessing techniques such as normalization, CLAHE, and pseudo-coloring enhance grayscale MRI scans for AI analysis. Simulated thermal imaging, using models such as the Pennes’ bioheat transfer equation, enables the creation of pseudo-thermal maps from grayscale MRI. These transformations help quantify temperature distribution, enabling non-invasive physiological assessment.

It covers key stages: thermal data collection via infrared thermography or MRI; preprocessing and temperature modeling; deep learning with CNNs and hybrid models; and final assessment through tumor detection, segmentation, and interpretability analysis.

### 3.1 Preprocessing and enhancement techniques

Thermal images, whether acquired via infrared thermography or generated from MRI, must be preprocessed to ensure consistent quality and to enhance features relevant to AI models.

- Normalization: Standardizing pixel intensity ranges for consistency across datasets (Wahab et al., 2015).
- Noise reduction: Filtering out acquisition artifacts and background interference to enhance signal-to-noise ratio (Vardasca et al., 2019).
- Contrast Limited Adaptive Histogram Equalization (CLAHE) improves local contrast in low-intensity regions, which is particularly important for detecting subtle tumor boundaries (Barnawi et al., 2021).

**Figure 4:**
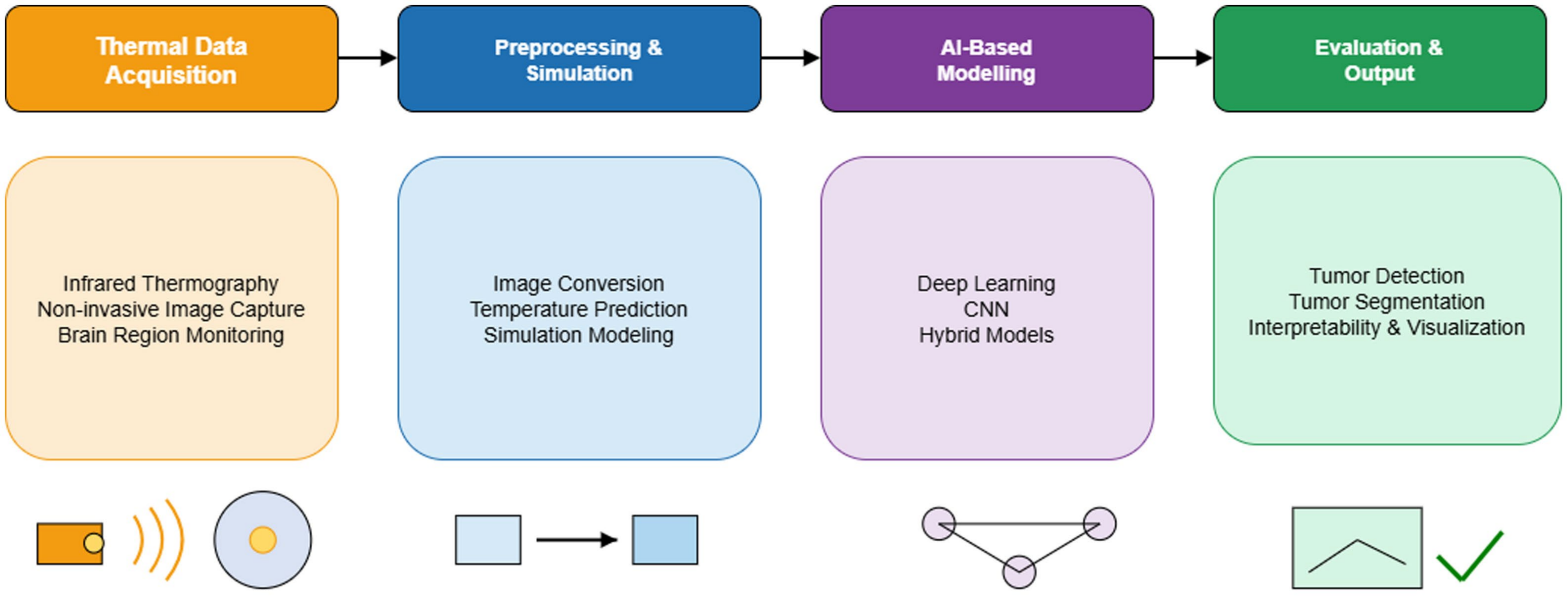
Overview of the AI-driven thermal imaging pipeline for brain tumor analysis (Kaifi, 2023).

These steps enhance visual clarity and substantially improve AI model performance, as illustrated in Figure 4. Research indicates that preprocessing pipelines with CLAHE and denoising can raise Dice similarity scores for segmentation models from 0.85 to over 0.90 (Gershenson and Gershenson, 2023).

### 3.2 Pseudo coloring and RGB conversion

**Figure 5:**
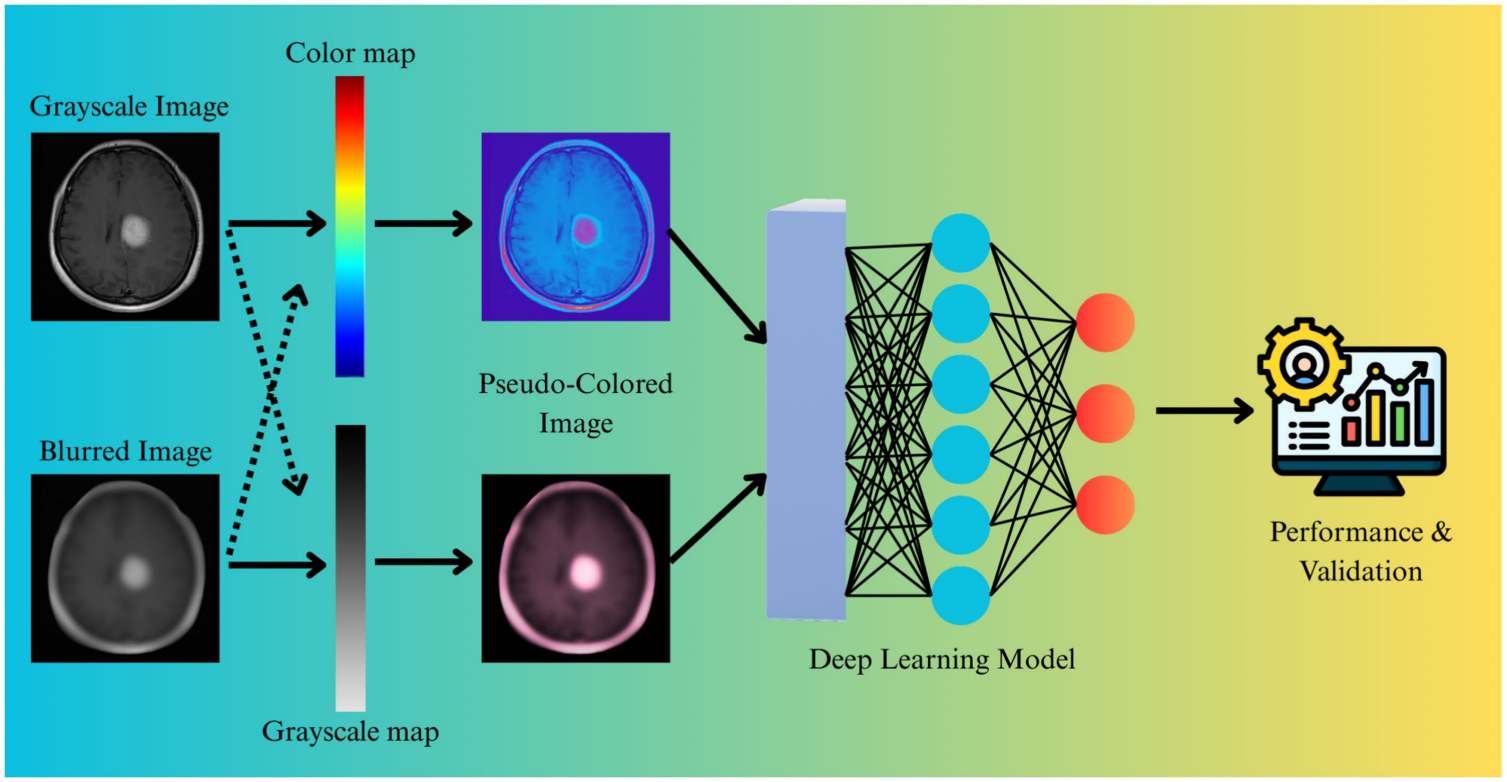
Applying pseudocoloring to grayscale medical images enhances visual interpretability and facilitates transfer learning with deep learning models (Krishnan et al., 2024). (Icon reproduced with permission from www.freepik.com: “Human resources icon”, https://www.freepik.com/icon/human-resources_15165779, created by zero_wing).

Many state-of-the-art deep learning models were originally developed for RGB images. To utilize pre-trained architectures, grayscale MRI or thermal images are often converted using pseudo-coloring techniques. Color mapping (e.g., “jet” or “hot” colormaps) assigns specific colors to certain intensity ranges, enhancing visual differentiation between tumor and healthy tissue (Ng, 2009). This enables transfer learning from large-scale natural image datasets, thereby accelerating model convergence, as illustrated in Figure 5. Several studies have shown that CNNs trained on pseudo-colored thermal images achieve higher classification accuracy than those trained on grayscale inputs, as described in Figure 5 (Etehadtavakol et al., 2019).

### 3.3 Simulated thermal mapping from MRI

When direct thermal acquisition is not possible, simulated thermal maps can be created using the Pennes’ bioheat transfer equation. This model includes:

- Metabolic heat generation from tumor cells,
- Blood perfusion rates that influence heat dissipation (Yahara et al., 2003),
- Thermal conductivity of tissues (Knapp et al., 2022; Ring and Ammer, 2012).

By applying these parameters to MRI voxel-intensity data, researchers generate pseudo-thermal maps that approximate physiological heat patterns. Although validation studies report low mean absolute errors (≈0.5 °C), our comparative review shows that such results are typically obtained in controlled settings. In real-world settings, inter-patient variability, acquisition inconsistencies, and anisotropic conductivity can reduce the accuracy of thermal maps. This emphasizes the need for cautious interpretation when using these simulations in a clinical setting.

### 3.4 Integration into AI pipelines

After preprocessing and simulation, thermal images are integrated into deep learning pipelines. Standardized and color-enhanced images are fed into convolutional layers for feature extraction. Hybrid architectures process both MRI and thermal channels, capturing structural and functional cues simultaneously. Studies have reported classification accuracies exceeding 95% when using combined MRI and thermal inputs processed through optimized CNNs (Owens et al., 2020; Aloraini et al., 2023).

While preprocessing techniques and bioheat-based simulations can boost feature extraction and improve thermal characterization, their robustness remains strongly influenced by modeling assumptions and acquisition variability in data collection. To mitigate this, we suggest focusing on patient-specific modeling and establishing standardized validation protocols in future studies to better ensure that simulations represent intraoperative conditions accurately.

## 4 Deep learning models for brain tumor detection

**Figure 6:**
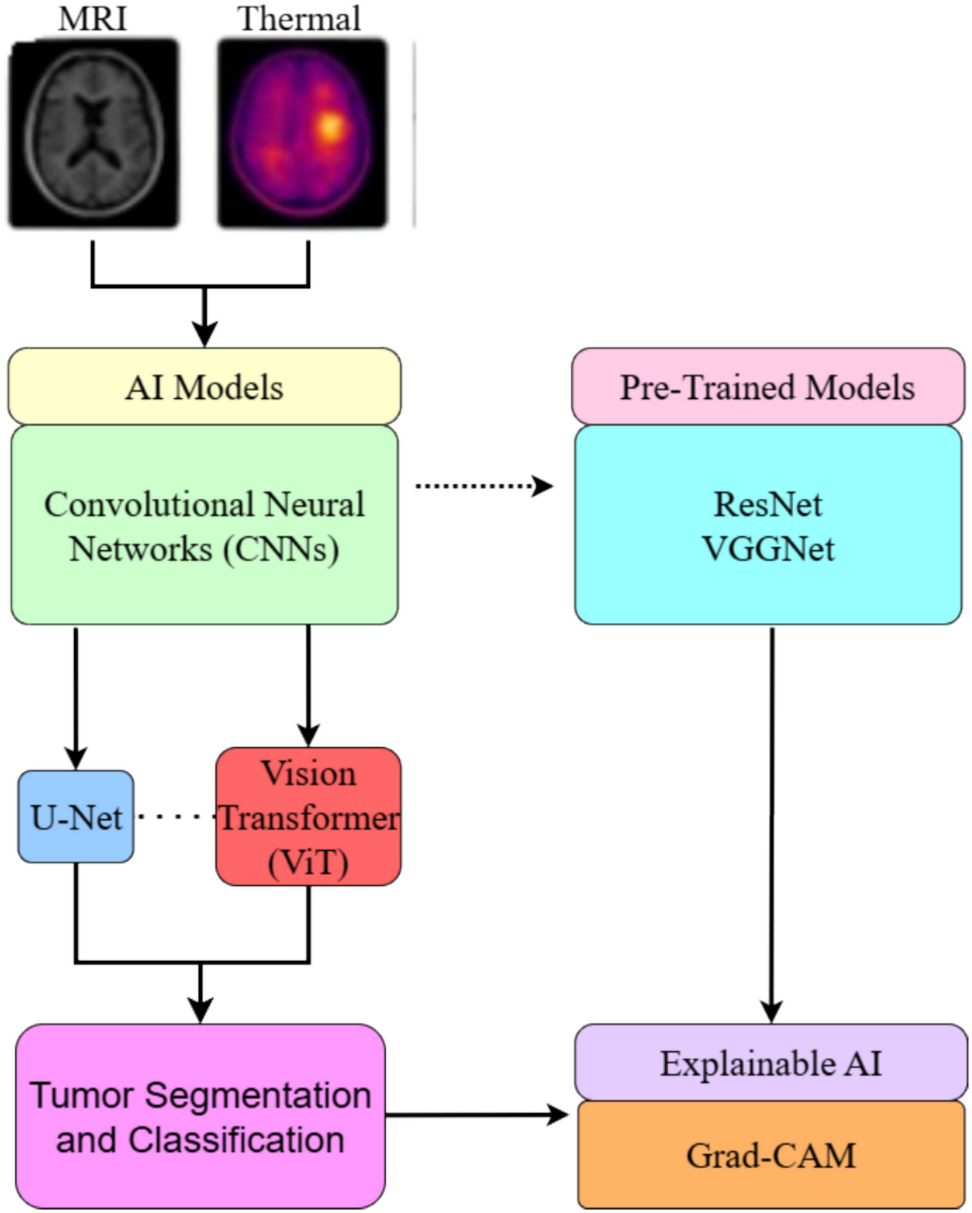
Illustrates how AI models such as CNNs, U-Net, and ViT, as well as pre-trained architectures, assist in brain tumor segmentation and classification, with Grad-CAM enhancing interpretability (Ullah and Kim, 2025). Licensed under the terms and conditions of the Creative Commons Attribution (CC BY) license (https://creativecommons.org/licenses/by/4.0/).

AI models, especially convolutional neural networks (CNNs), U-Net, and Vision Transformers (ViT), are used to segment and classify brain tumors from MRI and thermal images. Pre-trained models, such as ResNet and VGGNet, are often adapted through transfer learning, as shown in Figure 6. Hybrid models that combine multiple architectures further improve accuracy. Explainable AI techniques, such as Grad-CAM, enhance interpretability for clinical use. Figure 6 provides an overview of these models.

### 4.1 Convolutional neural networks and feature extraction

Deep learning has transformed medical image analysis, and CNNs remain the most effective methods; several models have been adopted for detecting brain tumors. CNNs automatically learn hierarchical features, enabling them to distinguish subtle differences in tissue texture, shape, and thermal patterns. Classic architectures such as VGG16, ResNet50, and DenseNet201 have been fine-tuned for medical imaging tasks, leveraging transfer learning from large natural image datasets to overcome the limited size of clinical datasets (Karagoz et al., 2024; Owens et al., 2020; Asiri et al., 2024).

When applied to MRI and thermal data, CNNs have achieved classification accuracies above 95% for differentiating between glioma, meningioma, and pituitary tumors (Aloraini et al., 2023; AlTahhan et al., 2023). It is worth noting that most CNN-based studies reviewed did not adequately address the impact of dataset imbalance or domain shift when using transfer learning. Based on our assessment, this may inflate performance metrics and necessitate external validation to confirm clinical utility.

### 4.2 Hybrid and ensemble architectures

To further enhance performance, researchers have developed hybrid models that integrate multiple architectures. For example, DRMv2Net, which combines DenseNet201, ResNet101, and MobileNetV2, extracts complementary features across different scales and depths, thereby improving sensitivity and specificity in brain tumor classification (Aloraini et al., 2023). Ensemble methods, which combine predictions from multiple models, also help reduce overfitting and improve robustness across various imaging datasets (Appiah et al., 2024; Alzubaidi et al., 2021).

### 4.3 Segmentation networks: U-net and variants

Accurate tumor localization is crucial for surgical planning and treatment monitoring. U-Net, with its encoder–decoder architecture and skip connections, has become the standard for medical image segmentation (Archana and Komarasamy, 2023). Variants such as U-Net++ and ResUNet incorporate residual connections and dense skip pathways, improving boundary detection and performance in low-contrast regions (Ali et al., 2022). When trained on preprocessed thermal-MRI datasets, U-Net-based models have achieved dice similarity coefficients (DSC) exceeding 0.90, indicating high overlap between predicted and ground-truth tumor masks (Gershenson and Gershenson, 2023; Ayadi et al., 2021).

### 4.4 Transformer-based models

Recent studies examine Vision Transformers (ViTs) and hybrid CNN-Transformer models, which capture long-range dependencies and global context. These models have demonstrated promising performance in brain tumor detection, particularly when combined with thermal information to enhance feature representation (Krishnan et al., 2024; Khan et al., 2023). Although Vision Transformers deliver impressive accuracy, we found that their high computational demands and need for large amounts of data limit their use in low-resource clinical settings. This reveals a trade-off between model complexity and ease of deployment that future research will need to address.

### 4.5 Explainable AI and interpretability

A major challenge in adopting AI in clinical settings is interpretability. Grad-CAM and related saliency methods have been integrated into CNNs and Transformers to generate heatmaps that highlight the regions that most contributed to a decision (Alzubaidi et al., 2021; Kumar et al., 2023). This transparency builds clinician trust and meets regulatory requirements for AI-assisted diagnostics.

### 4.6 Emerging clinical applications

Real-time platforms such as MediScan and similar AI-powered diagnostic systems demonstrate how these models can be integrated into workflow environments. These systems integrate multimodal imaging inputs, rapid inference, and user-friendly interfaces, providing decision support during diagnosis or intraoperative assessment (Anaya-Isaza et al., 2023).

This work is a narrative review that brings together findings from previously published studies. The training setups for the AI models discussed vary and are often not fully described. Many of the methods in Tables 1, 2 employ transfer learning with ImageNet-pretrained models such as ResNet, VGG, and Vision Transformer. Some models are built and trained from the ground up using MRI datasets specific to the field. Key details such as optimizer type, learning rate schedule, batch size, number of training epochs, and weight initialization are often missing from the original reports. This lack of detail makes it difficult to reproduce results and compare studies accurately. For this review, we present the model architectures and performance metrics as reported in each publication, noting that the training and optimization methods differ widely between studies.

**Table 2:** Comparison of deep learning models for brain tumor detection using MRI, thermal, or combined modalities.

Study	Model type	Input modality	Accuracy (%)	F1-score
35	CNN (custom)	Simulated Thermal + MRI	95.4	0.94
36	Transformer + CNN	MRI	97.2	0.96
38	Hybrid CNNs	MRI	96.1	0.95
37	Swin Transformer	MRI + Thermal	95.9	0.95
42	Deep CNN	MRI	93.5	0.91
7	Federated and Transfer Learning	MRI	94.8	0.93
6	Rotation-Invariant ViT	MRI	96.7	0.94
13	TransBTS (Transformer + 3D U-Net)	Multimodal MRI	95.3	0.92

Metrics include classification accuracy and F1-score, highlighting the advantages of hybrid and multimodal approaches.

The performance of various deep learning architectures in brain tumor detection is summarized in Table 2, which compares model types, input modalities, and key metrics across recent studies.

Beyond quantitative performance metrics, the AI methodologies reviewed in this section have tangible clinical relevance. Convolutional neural networks and transformer-based architectures can enhance radiological workflows by enabling earlier tumor detection and more reliable subtype differentiation. Similarly, segmentation frameworks, particularly U-Net variants, may support preoperative planning by improving delineation of tumor margins and peritumoral regions. Multimodal approaches that integrate thermal imaging with MRI offer additional value by incorporating physiological and metabolic signatures that extend beyond purely structural information, thereby potentially improving malignancy characterization. In intraoperative settings, real-time AI-driven thermal analysis could further assist surgical decision-making by identifying hypermetabolic zones and supporting more precise resection strategies. Nonetheless, widespread clinical adoption requires prospective validation, harmonized acquisition and preprocessing protocols, and appropriate regulatory clearance to ensure safety, robustness, and demonstrable clinical benefit.

The reported performance metrics are promising. However, increasing architectural complexity does not automatically translate into clinical utility. In our view, the real value of these AI strategies lies in their ability to combine multimodal physiological data while maintaining interpretability and practical deployment. Future research should emphasize generalizability and real-world robustness rather than focusing solely on small gains in accuracy.

## 5 Malignancy prediction using thermal behavior

### 5.1 Thermal physiology of malignant tumors

Malignant tumors differ from benign lesions not only in their growth patterns but also in their physiological heat signatures. Rapidly proliferating cells generate more metabolic heat, and abnormal angiogenesis alters local blood flow. These mechanisms create regions of hyperthermia near invasive margins or, conversely, hypothermia in necrotic tumor cores (Rezaei, 2025; Raghavapudi et al., 2021; Cleveland Clinic, 2023). Studies using intraoperative thermography have documented temperature deviations of several degrees Celsius between tumor tissue and surrounding parenchyma, with hyperthermic rims often correlating with higher microvessel density (Chaichana and Quiñones-Hinojosa, 2019; Knapp et al., 2022; Yahara et al., 2003).

### 5.2 Mechanistic insight of brain tumor for AI-thermal imaging

Mechanistic insight explains why specific patterns arise in biomedical data by connecting AI outputs to biological and physical processes. In brain tumor imaging, altered physiological conditions create thermal signatures: tumors disrupt vascularization, increase metabolic activity, and modify perfusion dynamics, leading to localized heat variations. Malignant areas typically exhibit higher temperatures due to hypermetabolism and increased blood flow, whereas necrotic or hypoxic regions are cooler (Ghorbian et al., 2024). The Pennes bioheat equation indicates that metabolic heat generation in tumors can be 2.5 to 60 times higher than in normal tissue, with perfusion up to 50-fold higher, supporting the use of thermal imaging as a diagnostic tool (Shao et al., 2023). Without mechanistic validation, AI models risk overfitting to dataset artifacts that may lack clinical relevance. Intraoperative infrared imaging has shown temperature variations of ±0.5 to 2 °C in tumors, depending on tumor type and depth, highlighting physiological effects and potential confounding factors, such as anesthesia and tumor morphology (Sadeghi-Goughari and Mojra, 2015; Gorbach et al., 2004). By integrating mechanistic understanding into AI frameworks, we can improve model interpretability, foster clinician trust, and ensure predictions are both biologically plausible and clinically relevant.

### 5.3 AI-based classification of malignancy

Deep learning models trained on thermal-enhanced imaging datasets can utilize these physiological patterns. CNN-based vision transformers and hybrid models have been developed to distinguish benign from malignant tumors, achieving classification accuracies over 95% and F1 scores above 0.93 (Owens et al., 2020; Aloraini et al., 2023; AlTahhan et al., 2023). For example, ensembles combining ResNet and DenseNet on pseudo-thermal MRI demonstrated strong performance across multi-institutional datasets, highlighting the advantage of integrating functional cues into classification frameworks (Asiri et al., 2024; Alzubaidi et al., 2021).

### 5.4 Limitations and validation of simulated thermal imaging

While computational and simulated thermal imaging techniques are widely used for exploratory studies and model development in brain tumor research, their applicability is limited by significant practical and physiological constraints. Many current models may not fully capture the intrinsic heterogeneity of tumor tissue because they rely on oversimplified representations of blood perfusion and tissue characteristics (Ahmed et al., 2024; Yurtsever et al., 2024; Raghavapudi et al., 2021). In clinical settings, discrepancies between simulated predictions and *in vivo* measurements may arise from additional factors, such as cortical swelling, exposure to cerebrospinal fluid, and intraoperative tissue manipulation, which can further alter thermal patterns. Therefore, simulation-based thermal models must be thoroughly validated against direct intraoperative thermographic data before they can be reliably integrated into clinical or surgical workflows.

### 5.5 Tumor boundary estimation

Thermal gradients are also used safely to characterize invasive tumor margins. Simulation-based thermal maps derived from MRI data help identify transitional zones between healthy and tumor tissue, providing a non-invasive way to define resection boundaries (Ring and Ammer, 2012; Ng, 2009). AI segmentation models trained on this multimodal data have shown improved Dice scores, particularly for infiltrative tumor types (Gershenson and Gershenson, 2023; Ali et al., 2022).

### 5.6 Remaining limitations

Despite these advancements, challenges remain:

- Variability: Based on our review, patient-specific anatomical characteristics (e.g., skull thickness, vascular abnormalities) and ambient conditions (e.g., room temperature, sensor distance) introduce noise into thermal profiles. These confounders are often not accounted for in training pipelines, leading to inflated performance on controlled datasets but reduced generalizability in practice.
- Data scarcity: Few public datasets include paired thermal and histopathology labels (Wahab et al., 2015).
- Clinical validation: Most studies remain retrospective, requiring prospective trials for regulatory approval (Pedada et al., 2023).

Nonetheless, predicting malignancy from thermal behavior, enhanced by deep learning, has become a valuable complement to traditional imaging, providing a functional dimension that enhances diagnostic confidence and supports treatment planning. Table 3 offers a thematic summary of the key studies reviewed, highlighting their technical methods and imaging modalities to demonstrate current research trends.

**Table 3:** Summary of AI-based brain tumor studies by model type and imaging modality.

Study	Primary focus	Technique used	Modality
35	Thermal simulation & classification	Simulated thermal + CNN	Simulated thermal + MRI
36	Hybrid transformer-CNN classification	Transformer + CNN	MRI
38	CNN-based classification	Hybrid CNNs	MRI
37	Transformer with thermal integration	Thermal + Swin transformer	MRI + simulated thermal
42	CNN classification & segmentation	Deep CNN	MRI
7	Federated transfer learning	Federated + transfer learning	MRI
6	Rotation-invariant transformer	ViT	MRI
13	Multimodal segmentation with transformer	3D U-Net + Transformer	Multimodal MRI
19	Temperature modeling with PBTE	Pennes’ bioheat equation (PBTE)	Simulated thermal
20	Medical thermal imaging principles	Infrared thermography (IRT)	IRT

Furthermore, simulated thermal maps derived from bioheat models, such as the Pennes equation, depend on several assumptions, including tissue homogeneity and simplified boundary conditions. These assumptions may not hold in the context of complex, heterogeneous tumor environments. Consequently, discrepancies may arise between simulated and directly measured temperatures via intraoperative thermography, particularly in regions with necrosis or abnormal vasculature. These physiological limitations and potential sources of error must be rigorously quantified before these simulations can be deemed reliable for critical clinical decision-making.

Table 3 presents an overview of the studies reviewed in the manuscript, classified by focus area, AI techniques used, and imaging modalities (e.g., MRI, simulated thermal, and IRT). This mapping provides a comprehensive overview of current research trends and technical approaches in AI-based brain tumor diagnostics.

Thermal-based malignancy prediction adds a valuable functional aspect to tumor characterization. However, clinical translation requires rigorous physiological validation and multicenter evaluation. We think that combining mechanistic modeling with explainable AI is essential, as it helps ensure that predictions are based on biological mechanisms rather than mere data correlations.

## 6 Evaluation metrics

To ensure clinical reliability, AI models for brain tumor analysis must be thoroughly evaluated using standardized metrics tailored to their specific tasks. Below, we categorize key performance measures by application area.

### 6.1 Tumor segmentation metrics

Precise delineation of tumor boundaries is essential for surgical planning and treatment monitoring. The most commonly used metrics include:

- Dice Similarity Coefficient (DSC): Measures the overlap between predicted and ground-truth masks (range: 0–1), with values >0.90 indicating excellent agreement in recent multimodal studies (Gershenson and Gershenson, 2023; Ali et al., 2022).
- Intersection over Union (IoU): Similar to DSC but more responsive to boundary errors, with state-of-the-art models reaching IoU ≥ 0.85 (Ayadi et al., 2021).
- Hausdorff Distance measures the greatest boundary shift (in mm). particularly important for resection planning (ideal: <2 mm; Gershenson and Gershenson, 2023).

Example: U-Net variants that combine MRI and thermal inputs report a median DSC of 0.91–0.93 for glioma segmentation (Ali et al., 2022), surpassing MRI-only models by approximately 8%.

### 6.2 Tumor classification metrics

For distinguishing benign from malignant or predicting subtypes, studies usually report:

- Accuracy: Ranges from 93 to 97% for hybrid CNN-Transformer models (Owens et al., 2020; AlTahhan et al., 2023).
- Precision/Recall: High precision (>0.94) lowers false positives, while recall >0.92 guarantees sensitivity to malignancies (Aloraini et al., 2023).
- F1-Score: harmonic mean of precision and recall; multimodal approaches achieve 0.93–0.96 (Yahara et al., 2003; Asiri et al., 2024).
- AUC-ROC: Critical for clinical adoption, with top models reaching 0.98 AUC (Appiah et al., 2024).

Key Insight: Models that combine thermal and MRI inputs achieve 5–7% higher F1 scores than models using a single modality (Aloraini et al., 2023).

### 6.3 Thermal prediction metrics

When evaluating simulated or AI-predicted temperature maps:

- Mean Absolute Error (MAE): Bioheat-based simulations achieve an MAE of 0.4–0.6 °C compared to intraoperative measurements (Knapp et al., 2022; Yahara et al., 2003).
- Root Mean Square Error (RMSE): Typically, less than 0.8 °C for maps derived from Pennes’ equation (Ring and Ammer, 2012).
- Spatial correlation: Pearson’s R > 0.88 between predicted and measured thermal gradients (Civilibal et al., 2023).

### 6.4 Model interpretability and reliability

Beyond numeric scores, clinical utility relies on:

- Saliency Maps: Grad-CAM heatmaps identify key regions, such as hyperthermic rims (Khan et al., 2023).
- Uncertainty Quantification: Bayesian DL methods offer confidence intervals for their predictions (Pillai et al., 2023).
- Failure case analysis is essential for regulatory approval, as it helps address issues such as misclassified necrotic cores (Kumar et al., 2023).

Although Grad-CAM is widely used, there is an inconsistency in how interpretability results are validated. Few studies incorporate user testing or clinician feedback, thereby limiting the practical utility of saliency maps. Addressing this gap is essential for gaining regulatory approval. While the reported accuracies and Dice coefficients are promising, they should be interpreted with caution given the reliance on small sample sizes and single-center datasets, which can lead to overfitting. High performance in retrospective tests does not guarantee strong clinical outcomes. Additionally, some models prioritize algorithmic accuracy over clinically significant endpoints, such as survival prediction and treatment response. Future research should focus on validating results using clinically relevant endpoints and multicenter cohorts to enhance translational relevance.

Standardized evaluation metrics often report high performance. However, clinical readiness cannot be inferred from retrospective accuracy alone. Future validation should extend to prospective clinical studies, use of clinically meaningful endpoints, and formal uncertainty quantification. This ensures reliability, fosters clinician trust, and supports regulatory approval.

## 7 Current challenges and research gaps

Although significant progress has been made in integrating artificial intelligence with thermal imaging for brain tumor detection, several challenges and knowledge gaps remain to be addressed before these methods can be used routinely in clinical practice.

### 7.1 Limited multimodal datasets

A significant challenge in the field is the limited availability of large, annotated datasets that include paired MRI and ground-truth thermal imaging data. Most current studies rely on simulated thermal maps rather than authentic infrared thermography, which limits the generalizability of the models employed (Knapp et al., 2022; Wahab et al., 2015; Pedada et al., 2023). Furthermore, variations in acquisition protocols, hardware specifications, and preprocessing pipelines hinder the harmonization of datasets (Karagoz et al., 2024; Rahman and Islam, 2023). The limited availability of multimodal datasets poses significant challenges because existing repositories vary widely. Public datasets can differ in MRI protocols (T1-weighted, FLAIR, contrast-enhanced), image resolution, scanner manufacturers, and preprocessing methods, which can affect model generalizability.

Additionally, tumor subtype and grade distributions often show imbalances, with gliomas typically overrepresented (Kareem et al., 2023). Inconsistent annotation standards further complicate matters, as some datasets use manual delineations while others rely on semi-automated or consensus-based masks. Consequently, AI models may perform well in internal validation but struggle when tested in independent settings. To address these issues, it is essential to use larger datasets, harmonized acquisition standards, balanced tumor-class representations, and standardized annotation guidelines. Without these measures, models risk overfitting, which can impair their performance across different institutions and hinder effective clinical use.

The effectiveness of AI-driven thermal imaging systems is often limited by dataset biases and inconsistent methodologies. Dataset bias arises when training groups do not represent the broader patient population. For instance, models developed using glioblastoma cases may struggle with rarer tumor subtypes or when demographic factors such as age, ethnicity, and comorbidities vary between institutions (Oakden-Rayner, 2020).

### 7.2 Variability in thermal signatures

Thermal patterns are influenced by factors beyond tumor biology, including patient anatomy (e.g., skull thickness), imaging conditions, and vascular comorbidities. These variations can produce misleading temperature profiles, making it difficult for AI models trained on small or homogeneous datasets to perform accurately (Rezaei, 2025; Cleveland Clinic, 2023; Reddy et al., 2024).

### 7.3 Limited clinical validation

Although high accuracy has been demonstrated in retrospective studies, the need for prospective clinical trials remains recognized. Validation of clinical effectiveness and adherence to regulatory standards requires testing the technology in real-world settings, particularly during intraoperative procedures or outpatient diagnostics (Khan et al., 2023; Kumar et al., 2023). Concerns regarding reproducibility across diverse populations are raised by the reliance of many studies on data from a single institution (Ren et al., 2024). Retrospective analyses often rely on high-quality images from controlled conditions, which may not reflect the variability in real-world patient demographics, tumor types, or imaging settings (Liu et al., 2021). This selection bias can inflate performance metrics, such as Dice coefficients or classification accuracies, and limit the generalizability of the findings. Moreover, retrospective validation overlooks workflow constraints and challenges during intraoperative procedures. To address these issues, future research should focus on prospective multicenter clinical trials that assess AI-thermal imaging systems in routine diagnostic and surgical settings. Such trials are essential for establishing clinical reliability, obtaining regulatory approval, and integrating these technologies into neuro-oncology practice.

Moreover, much of the existing evidence derives from retrospective analyses of curated datasets presented under idealized conditions that do not accurately reflect surgical environments. These datasets are often homogeneous, leading to inflated estimates of reported accuracy. In contrast, prospective validation tests algorithms in real time on uncurated patients across diverse clinical settings, capturing the variability of intraoperative workflows, patient demographics, and equipment conditions (Kelly et al., 2019). Multicenter prospective studies are essential because they demonstrate whether models can generalize across institutions with different imaging protocols and hardware. Without such validation, claims of clinical readiness based on retrospective results can overstate the maturity of current methods. Addressing this requires well-designed prospective studies and meaningful clinical endpoints such as surgical margin accuracy, reduced operative time, and improved postoperative outcomes that are relevant to clinicians and patients (Topol, 2019). Acknowledging this gap strengthens the field’s scientific rigor and clarifies the path toward regulatory approval and real-world implementation.

### 7.4 Explainability and regulatory barriers

Although interpretability tools such as Grad-CAM help visualize AI decisions, there remains skepticism remain skeptical of “black-box” models. Regulatory bodies increasingly demand explainable AI outputs and comprehensive documentation of model performance and biases before approval for clinical use (Alzubaidi et al., 2021; Pillai et al., 2023; Sedeeq, 2024). It is essential to validate explainability in brain tumor imaging using methods such as saliency maps, Grad-CAM, and SHAP. One approach involves aligning AI-generated heatmaps with histopathological findings to confirm that the highlighted areas correspond to the actual tumor boundaries. Conducting user studies with radiologists and neurosurgeons can help assess whether these outputs enhance trust and decision-making. Additionally, standardizing metrics for explainability, such as localization accuracy and faithfulness scores, is recommended. Integrating explainability validation into clinical trials will ensure that AI tools are both interpretable and actionable.

### 7.5 Resource constraints and hardware limitations

Despite significant technological advancements, the widespread adoption of AI-driven thermal imaging for brain tumor detection is constrained by hardware availability. Many methods require high-performance GPUs or specialized thermal cameras, which are often expensive and not universally accessible. Even in high-income countries, these resources are often confined to tertiary care centers, resulting in disparities in access to advanced diagnostic support (Wilson et al., 2018). The absence of standardized imaging protocols can lead to discrepancies in recorded data due to variations in thermal camera resolution, calibration methods, lighting conditions, and acquisition angles. This undermines reproducibility across institutions and complicates the establishment of clinical benchmarks. Thus, developing consensus guidelines for thermal data acquisition and reporting is crucial to harmonize research outcomes and enhance collaboration among centers.

In low- and middle-income regions, the deployment of AI thermal systems is constrained by limited financial resources, shortages of skilled personnel, and inadequate digital infrastructure; MRI access is also limited, and high-resolution thermal cameras may not be available (Cazap et al., 2016). Deploying AI solutions in such environments requires optimizing for affordable hardware, software (e.g., MATLAB, Python), and lightweight models that can run on constrained systems without compromising diagnostic performance (Ullah and Kim, 2025; Reddy et al., 2024; Shamshad et al., 2024). The lack of portable, affordable, and energy-efficient solutions may limit adoption of these technologies to well-funded centers, thereby exacerbating global healthcare inequalities. To address this, we need cost-effective hardware platforms, streamlined acquisition processes, and international partnerships for technology transfer and training (World Health Organization, 2021).

In summary, AI-enhanced thermal imaging holds considerable promise for improving the diagnosis of brain tumors; however, several challenges remain. These include formalizing data-sharing protocols, strengthening model validation, validating results across multiple centers, and adhering to regulatory guidelines. To transition from promising research to functional, reliable clinical tools, these issues must be addressed.

We believe that the primary challenge to clinical integration lies not in the model’s performance but in the methodological limitations of current investigations. Although reported accuracy metrics are encouraging, progress remains constrained by the scarcity of well-curated multimodal datasets, the lack of prospective clinical validation, and inconsistencies in data acquisition and preprocessing practices. Resolving these structural limitations is more critical than introducing minor refinements to network architectures. Future efforts should prioritize coordinated data-sharing frameworks, standardized imaging workflows, multicenter validation studies, and clearly defined clinically meaningful endpoints. Without these essential measures, AI-assisted thermal imaging is likely to remain a promising research direction rather than a consistently dependable clinical tool.

## 8 Future research directions

The integration of AI with thermal imaging for brain tumor diagnosis holds significant promise; however, several key issues must be addressed to fully realize its clinical potential (Hossain et al., 2024; Heckel et al., 2024). Based on current limitations and emerging opportunities, we prioritize future research directions into three key categories:

A Immediate Priorities (0–3 year horizon)

I Standardized Multimodal Datasets

- *Rationale:* The main obstacle to model generalization is the absence of large, diverse datasets that combine thermal imaging with MRI/CT and histopathological confirmation.
- *Recommended Actions*:
  ■ Establishment of international repositories with standardized acquisition protocols
  ■ Development of open-access benchmarks similar to BraTS for thermal-enhanced tumor analysis

II Prospective Clinical Validation

- *Rationale*: While retrospective studies demonstrate promising accuracy (Section 6), real-world clinical performance remains unproven.
- *Recommended Actions*:
  ■ Multicenter clinical trials comparing AI-thermal diagnostics against current gold standards
  ■ Development of regulatory pathways specific to thermal-AI diagnostic devices

B Transformative Innovations (3-5 year horizon)

III Physiologically Informed Thermal Modeling

- *Current Limitations*: Pennes’ bioheat equation oversimplifies tissue heterogeneity and dynamic perfusion changes.
- *Proposed Solutions*:
  ■ Patient-specific models incorporating CFD and individualized vascular mapping
  ■ Machine learning-enhanced solvers for real-time intraoperative simulation

IV Dynamic Functional Thermography

- *Emerging Opportunity*: Temporal thermal patterns may provide insights into tumor metabolism and treatment response.
- *Research Directions*:
  ■ Development of dedicated temporal deep learning architectures
  ■ Investigation of thermal recovery patterns following provocation tests

V Validation Data of Tumor Properties and Characteristics

- *Current Limitation*: Tumor properties and characteristics may provide insight into segmenting and classifying brain malignancies.
- *Research Directions*:
  ■ Classifying tumor properties with pixel intensity for reference to segment and locate the tumor
  ■ Investigation into malignancy for diagnosis and treatment process

C Foundational Enablers (5 + year horizon)

VI Explainable AI Frameworks

- *Clinical Need*: Current models lack sufficient interpretability for widespread clinical adoption.
- *Development Priorities*:
  ■ Integration of attention mechanisms with thermal saliency mapping
  ■ Uncertainty quantification for clinical decision support

VII Global Health Implementation

- *Equity Consideration*: Most current systems require expensive imaging infrastructure.
- *Innovation Pathways*:
  ■ Development of low-cost, portable thermal imaging systems
  ■ Federated learning approaches for privacy-preserving model development

### 8.1 AI technology using hardware for real-time clinical use

Real-time diagnostic performance is crucial for decision-making during surgeries. While Graphics Processing Units (GPUs) are commonly used in AI applications, their widespread availability and extensive software ecosystems (e.g., CUDA and PyTorch) introduce drawbacks such as high power consumption, heat generation, and limited portability (Sze et al., 2017). Field Programmable Gate Arrays (FPGAs) are a promising alternative for accelerating AI models in medical imaging, but they require specialized hardware programming expertise. Staffing challenges in clinical environments with limited IT infrastructure can be addressed by leveraging technology effectively. GPUs are well-suited for training machine learning models, whereas FPGAs are better suited for low-resource or portable applications. A hybrid approach that uses GPUs for training and FPGAs for optimized inference offers an effective way to integrate these technologies into clinical practice. FPGAs have reconfigurable architectures that can be optimized for specific tasks, such as segmentation and classification, thereby enabling low-latency inference and reduced energy consumption, making them suitable for operating environments (Venieris et al., 2019).

Studies show that FPGA-based implementations can efficiently accelerate convolutional neural networks and clustering algorithms, achieving accuracy comparable to or exceeding that of GPUs while using significantly less energy. In brain tumor imaging, FPGA systems can enable real-time processing of thermal data, tumor segmentation, and malignancy prediction during neurosurgery (Xiong et al., 2021). Furthermore, their compact size makes them ideal for low-resource clinical settings.

Integrating FPGA acceleration into AI-driven thermal imaging could bridge the gap between laboratory research and clinical applications, particularly in settings where portability and cost-efficiency are essential. Key considerations include the following:

- Limitations of GPU-Based Deployment
  - High power consumption and heat generation limit their application in intraoperative or portable settings.
  - The integration into compact systems is made difficult by their large physical footprint.
  - The widely available GPUs may not meet real-time latency requirements for surgical decision-making.
- Advantages of FPGA Acceleration
  - Low Latency and Energy Efficiency: Real-time processing of thermal data during surgery is facilitated.
  - Reconfigurable Architecture: Optimization for specific AI tasks, such as segmentation and classification, is supported.
  - Portability: Integration into compact, resource-constrained systems is easier than with GPUs.
  - Clinical Utility: Immediate estimation of tumor boundaries is enabled, making it particularly useful for intraoperative feedback.
- Challenges of FPGA Integration
  - Higher initial development costs are associated with non-GPU solutions.
  - Specialized programming skills are required, and longer development timelines are anticipated.
  - A limited number of ready-to-use medical imaging libraries is available compared to those for GPUs.
- Future Research Priorities
  - Performance comparisons between FPGAs and GPUs are conducted across latency, accuracy, and cost-effectiveness.
  - FPGA-based portable diagnostic systems are explored for use in low-resource clinical settings.
  - Existing MRI and thermal acquisition devices are integrated with FPGA platforms to enable real-time deployment at the bedside.

It is noted that while GPUs are the current standard, FPGAs have significant potential for next-generation AI-driven thermal imaging systems, particularly in intraoperative and resource-constrained settings.

### 8.2 Standardizing and validating multimodal data fusion, ensemble learning, and explainable AI

The clinical application of AI-driven thermal imaging frameworks is recognized as requiring not only innovative algorithms but also systematic guidance for standardization and validation. It has been demonstrated that multimodal data fusion, integrating MRI, spectroscopy, PET, and thermal imaging, can improve performance; however, reproducibility remains challenging because of variability in acquisition protocols, preprocessing methods, and annotation standards across institutions (Ghorbian et al., 2024; Kareem et al., 2023). To ensure the comparability and reproducibility of results across clinical sites, it is essential to establish harmonized protocols and open-source benchmarks.

Ensemble learning strategies combine predictions from multiple models to reduce overfitting and improve generalization (Abdar et al., 2021). However, for clinical adoption, clear validation frameworks are needed, including independent external datasets and prospective multicenter studies to assess diagnostic accuracy and clinical outcomes, such as surgical margin delineation and patient survival (Reyes et al., 2020).

Explainable AI (XAI) must move beyond basic visualization tools to standardized validation protocols, as clinician trust depends on alignment with biological and pathological realities (Amann et al., 2020). Incorporating quantitative evaluation metrics, such as localization accuracy, robustness, and faithfulness, into clinical trials will provide objective measures of trustworthiness (Tonekaboni et al., 2019). Future international collaborations and multi-institutional consortia will be crucial for establishing standards and enabling the safe and effective clinical translation of multimodal, ensemble-based, and explainable AI systems for brain tumor detection.

In summary, future research should bridge the gap between laboratory innovation and clinical use by emphasizing data quality, simulation accuracy, interpretability, and real-world testing. Through interdisciplinary collaboration, these efforts can enable AI-driven thermal imaging to become a common tool in brain tumor diagnosis. Alongside improvements in AI algorithms, the use of high-performance computing platforms, particularly Field-Programmable Gate Arrays (FPGAs), has shown considerable promise for enhancing the accuracy and real-time capabilities of brain tumor detection systems (Xiong et al., 2021). FPGAs are well suited to accelerating computationally intensive tasks in AI, such as thermal data preprocessing, tumor segmentation, and malignancy classification, because of their built-in features, including parallel processing, low latency, and energy efficiency (Almomany et al., 2024b). Several studies (Almomany et al., 2023; Almomany et al., 2024a; Almomany et al., 2022a; Almomany et al., 2022b) have shown that using FPGAs to build deep learning models or hybrid algorithmic pipelines can significantly improve accuracy and introduce desirable real-time system capabilities. This is particularly effective when accuracy and timing are significant factors. Furthermore, FPGA features enable the implementation of more complex frameworks that integrate multiple algorithms and multiple layers of authentication to enhance overall performance (Jarrah et al., 2022), which can be effectively applied to next-generation neuro-oncology diagnostics.

## 9 Conclusion

This review is not merely a literature review; it presents a critical synthesis grounded in our multidisciplinary expertise in biomedical imaging, AI model development, and thermal simulation. In several sections, we highlight gaps between laboratory performance and clinical usefulness, particularly when simulated thermal data diverge from the real-world physiological complexity. We emphasize the importance of prospective validation, resource-aware implementation, and rigorous assessments of interpretability. Through this review, we aim to guide future research and promote the practical and equitable use of AI-powered neurodiagnostic tools.

Early and precise detection of brain tumors is essential for improving patient outcomes. However, conventional imaging modalities such as MRI and CT are limited by high costs, restricted access, and a focus on structural rather than functional information (Krishnan et al., 2024; Ahmed et al., 2024; Ullah and Kim, 2025). Recent developments in artificial intelligence and thermal imaging, including infrared thermography and bioheat-based simulation, offer promising alternatives by capturing functional signals related to tumor metabolism and vascularity (Rezaei, 2025; Cleveland Clinic, 2023; Knapp et al., 2022).

Deep learning architectures, including convolutional neural networks (CNNs) and transformer-based models, have demonstrated strong performance in tumor classification, lesion segmentation, and even temperature distribution prediction with clinically relevant accuracy (Owens et al., 2020; AlTahhan et al., 2023; Khan et al., 2023). Incorporating thermal information enhances the model’s discriminative ability, enabling more sensitive detection of malignancies and more precise delineation of tumor boundaries for treatment planning (Ring and Ammer, 2012; Gershenson and Gershenson, 2023; Ali et al., 2022).

Nonetheless, several challenges impede clinical adoption, including the lack of multimodal datasets, variability in thermal signatures, and the absence of prospective validation studies. Future research should focus on large-scale data sharing, advanced simulation techniques, multimodal data fusion, and explainable AI methods to meet regulatory and clinical standards (Pedada et al., 2023; Pillai et al., 2023; Sedeeq, 2024). Additionally, adapting these technologies for use in low-resource settings is essential to maximize their global health impact (Ullah and Kim, 2025; Reddy et al., 2024).

AI-powered thermal imaging offers a unique opportunity to integrate anatomical and functional tumor analysis, enabling non-invasive, cost-effective diagnostic solutions. Advances in multimodal AI integration and personalized thermal modeling will be crucial to the widespread clinical adoption of these technologies.

In summary, AI-enhanced thermal imaging represents a significant advance in brain tumor diagnostics, shifting the field from purely anatomical assessment to comprehensive functional-structural analysis. Through ongoing interdisciplinary collaboration and clinical validation, these innovations have the potential to significantly improve diagnostic accuracy, reduce reliance on invasive procedures, and expand access to advanced neuroimaging technologies worldwide.
